# The Relationship between Phthalates and Diabetes: A Review

**DOI:** 10.3390/metabo13060746

**Published:** 2023-06-11

**Authors:** Melissa Mariana, Elisa Cairrao

**Affiliations:** 1 CICS-UBI—Health Sciences Research Centre, University of Beira Interior, Av. Infante D. Henrique s/n, 6200-506 Covilhã, Portugal; melissa.r.mariana@gmail.com; 2 FCS-UBI—Faculty of Health Sciences, University of Beira Interior, 6200-506 Covilhã, Portugal

**Keywords:** plasticizers, phthalates, di-(2-ethylhexyl) phthalate (DEHP), butylbenzyl phthalate (BBzP), di-butyl phthalate (DBP), gestational diabetes, type 1 diabetes mellitus, type 2 diabetes mellitus

## Abstract

Since the beginning of their production, in the 1930s, phthalates have been widely used in the plastics industry to provide durability and elasticity to polymers that would otherwise be rigid, or as solvents in hygiene and cosmetic products. Taking into account their wide range of applications, it is easy to understand why their use has been increasing over the years, making them ubiquitous in the environment. This way, all living organisms are easily exposed to these compounds, which have already been classified as endocrine disruptor compounds (EDC), affecting hormone homeostasis. Along with this increase in phthalate-containing products, the incidence of several metabolic diseases has also been rising, namely diabetes. That said, and considering that factors such as obesity and genetics are not enough to explain this substantial increase, it has been proposed that the exposure to environmental contaminants may also be a risk factor for diabetes. Thus, the aim of this work is to review whether there is an association between the exposure to phthalates and the development of the several forms of diabetes mellitus, during pregnancy, childhood, and adulthood.

## 1. Introduction

Used in the plastic industry since the 1930s, phthalates are the most common and ubiquitous plasticizers worldwide. These are man-made chemicals mainly used to provide flexibility and elasticity to rigid polymers, and are used as additives and solvents in pharmaceuticals, cosmetics, and personal care products [1]. Being easily released from the parent product and absorbed by the human body, many studies have been carried out over the last few decades to understand their role in human health, especially due to their endocrine disruptor properties. This feature is extremely important since these compounds have the ability to interfere with hormones, even at very low levels of exposure, leading to adverse health effects [1,2]. Thus, the exposure to phthalates has already been associated with organ damage, cancer, reproductive system malformations, abnormal neurodevelopment, hypertension, atherosclerosis, myocardial infarction, metabolic syndrome, and insulin resistance [1,3,4,5,6].

Insulin resistance, the abnormal response of the body’s tissues to insulin, is one of the main causes of diabetes mellitus [7], which is one of the most prevalent metabolic diseases in the world [6]. According to the International Diabetes Federation, the incidence of diabetes has been increasing over the last few years, predicting a prevalence of 700 million cases in 2045, globally [8]. So, it is imperative to reduce this tendency, and a possible strategy is to adapt the population’s lifestyle to reduce the risk factors. Among the commonly known risk factors (obesity, unhealthy lifestyle, and family history of diabetes), the exposure to environmental contaminants, such as phthalates, has been identified as a strong risk factor [6].

Considering that environmental exposure has been pointed out as a possible trigger for the several forms of diabetes mellitus, the aim of this review is to establish the possible link between the exposure to phthalates and the development of diabetes mellitus, during pregnancy, childhood, and adulthood. For that, bibliographic research was carried out in different databases (PubMed, Scopus, and Web of Science), restricted to the years 2017–2023, up until January. However, any publication prior to 2017 that was considered relevant to this topic was also included in this review. The inclusion criteria comprised epidemiological and experimental studies, in which the exposure to phthalates was directly and indirectly related to type 1, type 2, and gestational diabetes mellitus. The exclusion criteria included all articles that were in duplicate, unrelated, inaccessible, and not written in English.

## 2. Phthalates

Usually referred to as plasticizers, phthalates are environmental contaminants that are ubiquitous in our everyday life. According to the number of carbon atoms in the molecular structure, these compounds are chemically divided into high- and low-molecular-weight phthalates (HMW and LMW). The first group is mostly used as a plasticizer in toys, medical devices, food packaging, and household products, due to their flexibility and elasticity properties, and comprises di-(2-ethylhexyl) phthalate (DEHP), butylbenzyl phthalate (BBzP), diisononyl phthalate (DiNP), di-n-octyl phthalate (DnOP), and diisodecyl phthalate (DiDP). On the other hand, the LMW phthalates are added in solvents, inks, pharmaceuticals, cosmetics, and personal care products and include di-butyl phthalate (DBP), dimethyl phthalate (DMP), diethyl phthalate (DEP), and di-isobutyl phthalate (DiBP) [1,3,7].

Moreover, since they do not have covalent bonds, these compounds are easily released from the original products, being able to contaminate the environment and be absorbed by animals and humans. Once in the human body, they undergo metabolization to their respective monoesters, being mainly eliminated in the urine, but they have also been found in other biologic matrices [1,9,10,11,12,13,14].

Since they are considered as endocrine disruptor compounds (EDCs), they can interfere with hormone homeostasis, thus leading to impaired health effects [1,6,7]. For this reason, some countries already have regulations for the use of phthalates; however, due to their ubiquity, they are still present in our daily lives [1,15]. Thus, it is necessary to prohibit their use and/or teach the population about behaviors that can help to reduce their exposure.

## 3. Diabetes Mellitus

### 3.1. Gestational Diabetes Mellitus

Gestational diabetes mellitus (GDM) is one of the most common complications during pregnancy that, according to the International Diabetes Federation (IDF) Atlas, affected 16.7% of pregnancies worldwide in 2021 [8]. It is defined by the occurrence of hyperglycemia during pregnancy, with no signs of previous diabetes, which usually disappears after childbirth. Chronic insulin resistance and glucose intolerance due to pancreatic β-cell dysfunction are the main factors for this gestational glucose increase [16]. Moreover, some alterations have already been shown in genes related to insulin secretion and insulin receptors in GDM cases [17]. In addition to the complications that can occur during pregnancy, GDM is also associated with problems in maternal and offspring postnatal health, with increased risks for type 2 diabetes mellitus, obesity, metabolic syndrome, and cardiovascular diseases [16,18]. Maternal age, ethnicity, obesity, and diabetes family history are traditional factors associated with GDM [18]; however, a large proportion of women with GDM do not have these risk factors, and environmental contaminants have been suggested as a potential risk factor, considering the increased exposure to these chemicals [19,20].

### 3.2. Type 1 Diabetes Mellitus

Type 1 diabetes mellitus (T1DM) is an autoimmune disease that affects primarily children and adolescents. The incidence of T1DM has greatly increased in the last century, becoming the most common chronic disease in this young age group, with an annual incidence of 31,000 new cases per year in Europe and 150,000 worldwide [8,21,22]. The development of T1DM results from the targeting and destruction of pancreatic β-cells by the immune system, resulting in no (or insufficient) production of insulin [21,23]. The first signals of the disease, with the appearance of islet autoantibodies, normally start months to years before the clinical diagnosis, suggesting that besides the genetic and epigenetic factors, environmental exposure may also be involved in T1DM development, either pre- or post-natal exposure. In fact, it has already been suggested that in genetically susceptible subjects, the autoimmune response against β-cells is highly possible to be triggered by environmental factors [22,23,24]. This way, environmental contaminants, including phthalates, have emerged as a possible risk factor for T1DM.

### 3.3. Type 2 Diabetes Mellitus

According to the IDF, type 2 diabetes mellitus (T2DM) accounts for around 90% of all diagnosed diabetes, with a worldwide estimated occurrence of 536.6 million people [8]. In this chronic disease, hyperglycemia, that is, increased blood glucose levels, occurs due to insufficient amounts of insulin or ineffective insulin function. One of the main causes is insulin resistance, which is defined as the incapacity of the body cells to respond to normal or high levels of insulin. This will promote an increase in insulin production that, with time, leads to a failure and loss of the pancreatic β-cells, resulting in islet dysfunction and a consequent decrease in insulin production [8,25,26]. Thus, the dysfunction of β-cells is another reason for hyperglycemia occurrence. Although not completely understood, several factors have been known as major increasing risks for T2DM, including age, ethnicity/race, obesity, and genetic inheritance [8]. The genetic component has a high weight in the development of T2DM, and genes related to insulin secretion, β-cell function, development, and survival have already been linked to an increased probability of developing the disease [26]. There is also a close relationship between T2DM and obesity, as one can lead to the development of the other. Besides the elevated free fatty acid levels, other substances secreted by the adipocytes that are augmented (tumor necrosis factor—TNF-α, resistin) or decreased (adiponectin) in obesity inhibit insulin secretion and insulin-mediated glucose uptake, and lead to insulin resistance [26]. More recently, environmental factors have been suggested as a possible cause for T2DM, and, considering the already known role of phthalates in obesity as obesogens, there is a strong potential for these chemicals to affect T2DM.

## 4. Phthalates as a Risk Factor for Diabetes Mellitus

### 4.1. Gestational Diabetes Mellitus

#### 4.1.1. Epidemiological Studies

In the USA, three different studies using the same cohort, the LIFECODES pregnancy cohort, presented different outcomes. With the aim of analyzing the link between exposure to phthalates and risk factors for GDM, the authors quantified the levels of phthalates in the urine of 350 pregnant women and related them to first trimester body mass index (BMI), gestational weight gain (GWG), and second trimester glucose levels. The results showed a positive association between MEP and GWG and impaired glucose tolerance, and a negative one regarding MBP, MCPP, and ∑DEHP levels, and excessive GWG, continuous blood glucose, and impaired glucose tolerance, respectively [27]. When evaluating phthalates metabolites separately and combined in the first and second trimesters of pregnancy, the same research group found that phthalates and their mixtures may be involved in maternal glucose metabolism, since in the first trimester there was a negative correlation between MBP, MCNP, and MCPP levels and GDM and impaired glucose tolerance, while a positive association was found for MiBP and MHBP levels and impaired glucose tolerance and GDM, respectively. Moreover, the mixtures of phthalates presented similar results to the individual phthalate effects [28]. On the other hand, Noor et al. found no association between maternal urinary phthalate metabolites and infants’ birth weight from mothers with higher levels of glucose during pregnancy [29].

Reporting on a different cohort, similar results were found by Shaffer and colleagues, with urinary MEP levels being associated with GDM. In this study, 705 pregnant women provided one spot urine sample in the first and third trimesters, which were compared with GDM screening (performed between gestational weeks 24 and 28). Apart from the confirmed relationship between MEP and GDM, the levels of MBP and MCOP were associated with impaired glucose tolerance, and MCPP had a negative association with GDM. Moreover, the authors also found a possible link regarding race/ethnicity [30]. This matter must be studied further, but it seems to be in accordance with the numerous hypotheses regarding population variability.

In a different perspective, James-Todd et al. performed another prospective study, this time studying 245 pregnant women who attended a fertility clinic, where urinary DEP and DiBP metabolites (MEP and MiBP) were found to be increased and decreased, respectively, in women with higher glucose levels. It is of note that the sources of exposures of these two phthalates were predominantly different, with DEP being found in personal care products while DiBP was found in food and consumer products, and this was a subfertile population, with a higher risk of glucose dysregulation during pregnancy [31]. Reporting on the same cohort, Bellavia et al. aimed to understand the link between the use of personal care products containing phthalates and the occurrence of GDM. For this, 233 women answered a questionnaire regarding the use of personal care products (concerning the previous 24 h), and blood samples were collected at the end of the second trimester. The authors found a correlation between increased levels of blood glucose and bar soap, deodorant, and lotion, which, from other statements, are related to phthalates [19].

A longitudinal cohort involving 3269 women that provided urine and serum samples at each trimester of pregnancy found that early pregnancy exposure to phthalates may be involved with an increased risk of GDM. Specifically, higher urinary concentrations of MBP, MMP, MEOHP, and MEHHP were associated with increased blood glucose in the first trimester [32]. Three different Chinese case-control studies reported an association between phthalate exposure during pregnancy and the occurrence of GDM. Comparing women with and without GDM, Liang and colleagues also found a relationship with phthalate exposure, since there were higher levels of MEHP in the GDM cases. Moreover, MMP, MEP, MiBP, MECPP, and MEOHP have also been linked to fasting blood glucose and insulin, and insulin resistance index, which are parameters related to GDM [33]. A different cohort enrolled 676 women divided in two groups, with and without GDM, for whom urine samples were collected in early pregnancy. The urinary levels of MnOP, MBzP, MEOHP, and MECPP were all significantly associated with GDM; however, MEOHP was found to be independently linked to GDM at concentrations higher than 15.6 µg/L. Considering these results, almost 25% of the participants had an increased risk for GDM due to MEOHP concentrations [34]. More recently, relying on phthalate levels quantified in the serum of 201 women (at the time of delivery), Wang and colleagues found that MBP was the most abundant metabolite in this population sample, and they also showed a significant association between MBP and MiBP levels and the 2 h blood glucose, which in turn is related to GDM [35]. Thus, this study also shows a correlation between phthalate exposure during pregnancy and the occurrence of GDM. A different study also reported an association between phthalates and GDM in early pregnancy. Serum phthalate metabolites measured during 10–17 weeks of gestation (for women with singleton male pregnancies) showed a positive relationship between MiBP and GDM, and the quantification of the MEHP and MCOP of pregnant women without GDM was related to stimulated blood glucose levels [36].

In a different approach, Martinez-Ibarra et al. reported on a Mexican population of women with and without GDM. This time, the serum levels of three of the four evaluated miRNAs related to GDM were associated with urinary concentrations of different phthalate metabolites (MBzP, MBP, MEHP, and MiBP). It is important to note that almost 100% of the urine samples were positive for phthalate levels [37] and were several times higher than the ones reported in other studies [29,30]. These differences might be due to the different populations under study, considering that that Noor et al. and Shaffer et al. investigated American women while Martinez-Ibarra and colleagues investigated a Mexican cohort, which in turn is a country that has not yet regulated the use of phthalates, so the Mexican population is much more exposed to these types of products, and consequently at greater risk [37]. In a different Mexican cohort, 618 women provided urine samples in the second and/or third trimesters of pregnancy, which were then related to metabolic biomarkers in blood samples collected 4–5 and/or 6–8 years after delivery. In addition to the connection with some lipid parameters, the results showed a positive association between MECPTP and ∑DBP and increased glucose and insulin levels, insulin resistance, and glycosylated hemoglobin (HbA1c). These are very interesting findings, since a pre-natal exposure to phthalates seems be associated with long-term adverse health effects in the mother [38]. It has already been pointed out by other researchers that GDM, which may be due to phthalate exposure, can result in maternal and offspring health problems later in life [16,18]; however, these findings suggest that the exposure to these compounds during pregnancy, without causing any disturbance during this sensitive period, appears to be associated with metabolic changes in the future.

A different investigation also found an association between MBP, MiBP, and MEHP levels and GDM, though this study analyzed newborn exposure to phthalates in utero by quantifying their levels in meconium, and the association was only found for mothers of male fetuses [39].

Placental corticotropin-releasing hormone (pCRH), a placenta-produced neuropeptide that greatly increases during pregnancy, has been linked to hypertension in pregnancy, depression, and trauma. Bearing this in mind, and that exposure to phthalates can be even more harmful in pregnant women with pre-existing complications, a cohort of 1018 participants was gathered to find a negative association between phthalate mixtures and pCRH levels in women with GDM, particularly in the third trimester. These results suggest that phthalates affect the production of pCRH differently throughout pregnancy [40].

Although most studies show a correlation between exposure to phthalates and GDM, as seen in Table 1, other authors have obtained opposite results, with no association found with GDM nor any adverse glycemic outcomes for the phthalate metabolites analyzed [41,42,43]. It is necessary to take into account that differences throughout these epidemiological studies, whether due to their design, population, or biological samples, can affect the outcome; either way, more studies are needed to understand if and how phthalates affect GDM.

#### 4.1.2. Experimental Studies

Until now, there has only been one study performed in animals relating phthalates to GDM, which may be a window for the mechanistic pathways. In this study, the authors managed to induce GDM in Sprague Dawley rats with the administration of DBP and streptozotocin (STZ), a new and more relevant model for GDM. Moreover, in vitro and in vivo studies demonstrated that exposure to DBP led to FoxM1 downregulation by pSTAT1, resulting in the decreased viability and apoptosis of β-cells, culminating in GDM [44].

#### 4.1.3. Possible Mechanisms

Insulin resistance and inflammatory factors have been considered as the main factors responsible for GDM pathophysiology [45]. Yet, the increasing exposure to environmental contaminants has suggested phthalates as risk factors for several diseases, including GDM, either directly or by acting on GDM triggers. For instance, it is known that TNF-α is related to GDM by inducing adipocyte lipolysis, which can lead to a decreased insulin sensitivity by peripheral tissues, thus being considered as a biomarker for insulin resistance in pregnancy [35,46]. Using a network-based approach to understand the mechanism behind the link between DEHP and GDM, Zhang and colleagues found that exposure to DEHP may increase TNF-α expression, which suppresses GLUT4 (glucose transporter protein), as well as glucose uptake, disturbing glucose homeostasis and culminating in GDM [47]. In addition, phthalates have already been described to interact with peroxisome proliferator-activated receptors (PPAR), nuclear receptors related to glucose and lipid metabolism [48,49]. Of the main isoforms of these receptors, PPARα, is the one implicated in β-cell functioning, being responsible for insulin secretion. Thus, the interaction between phthalates and PPARα may disturb blood glucose homeostasis [28]. Moreover, PPARγ, which is related to adipogenesis, is also activated by phthalates, promoting obesity, which is an important factor for the occurrence of GDM [35]. Oxidative stress has also been suggested as a possible mechanism for GDM. In addition to being related to increased reactive oxygen species (ROS), phthalates and homeostasis model assessment-estimated insulin resistance (HOMA-IR) have been associated with a biomarker for oxidative stress (malondialdehyde—MDA) [35,50,51]. As the name implies, phthalates as EDCs may disrupt the endocrine system by interfering with the action of hormones [52]. Specifically, phthalates have been described as agonists of the estrogen receptors (ER), and estrogens are linked to insulin resistance; thus, phthalates can promote insulin signaling through ERα mediated pathways, which, when sustained, may lead to excess insulin release, β-cell exhaustion, and peripheral insulin resistance [30].

Although all of these have already been described as pathways for impaired glucose metabolism and insulin resistance, it is still unclear how phthalates promote the development of GDM; so, more studies are needed to unravel the actual mechanisms.

### 4.2. Type 1 Diabetes Mellitus

#### 4.2.1. Epidemiological Studies

Very few studies have linked phthalates to T1DM so far, mainly epidemiological ones, considering the modest incidence of the disease [53]. Nevertheless, one study performed in Portugal evaluated the urinary concentration of phthalates in children with new-onset and existing T1DM and controls. The authors found no significant association between phthalate levels in the T1DM cases compared to controls; however, there was a higher concentration of MiBP in children with new-onset T1DM [54]. Considering that this study relied on a small population sample, it is possible to hypothesize that resorting to a larger sample could have significant results for the relationship between phthalates and T1DM.

#### 4.2.2. Experimental Studies

In animal models, phthalates effects have been evaluated together with bisphenol-A (BPA). When exposing non-obese diabetic (NOD) mice to relevant human doses of BPA and a mixture of phthalates, it was found that phthalates did not accelerate the development of T1DM; in fact, phthalates seem to diminish the effects of BPA on the number and function of macrophages, but not in insulitis development. This possible hormesis effect (protective role) of phthalates may be due to the typical non-monotonic curve, in which high doses may decrease the development of diabetes [55]. A different study showed that phthalate metabolites have less capacity to affect insulin secretion and viability in the rat pancreatic β-cell line (INS-1E) than BPA [56]. Although it is important to study the effects of a mixture of EDCs, since human beings are exposed to several contaminants at the same time, these reports hamper the study of the relationship and mechanisms of action of phthalates alone in the development of T1DM.

There is a huge gap regarding EDCs’ effects on the development of T1DM. Therefore, in addition to the need for experimental studies to understand how phthalates affect β-cells, epidemiological studies with larger sample sizes are also essential to understand whether phthalates are really involved in the development of T1DM.

#### 4.2.3. Possible Mechanisms

Several pathogenic mechanisms have been pointed out as possible T1DM triggers by EDCs, including effects on β-cells, immunomodulation, epigenetics, microbiota, and vitamin D [21,53]. As previously shown, phthalates were already reported to directly affect rat β-cell secretion and viability [56]. Moreover, it is known that the activation of estrogen receptors can lead to glucose-induced insulin synthesis, its secretion by β-cells, and their survival from pro-apoptotic stimuli [53,57], and so considering that phthalates can act on these receptors [58], they can also indirectly affect β-cells through the estrogen receptors. EDCs may also affect the immune system by modulating the function of immune cells and cytokine levels, which may result in T1DM [21,53]. In experimental studies, pre-natal exposure to low doses of phthalates has been linked to epigenetic changes in genes related to the immune response in the offspring, which can promote autoimmunity [53,59]. In addition, the gut microbiota is important for a healthy immune system; however, a change in its composition has been associated with the development of T1DM [60]. Considering that phthalates have been found to alter the gut microbiota in a rodent model [21,61], it is a possible mechanism for T1DM promotion. Additionally, a vitamin D deficit and decreased intracellular calcium levels have also been related to T1DM [21,62], and, in turn, phthalates have been associated with changes in these two parameters. Specifically, urinary levels of phthalate metabolites were negatively related to circulating 25-hydroxyvitamin D [21,63,64], and phthalates have been involved in alterations in calcium handling levels, and calcium channel activity [5,65,66,67,68]. Thus, phthalates may be involved in T1DM development through vitamin D and calcium channel changes. Although all of these studies provide some evidence of the association between exposure to phthalates and T1DM and the possible mechanisms involved, more studies are needed, either experimental or epidemiological, to understand the actual effects of phthalates in this autoimmune disease.

### 4.3. Type 2 Diabetes Mellitus

#### 4.3.1. Epidemiological Studies

In order to analyze how pre-natal exposure affects metabolic risk factors during childhood, 757 children from women that provided urine samples during pregnancy (one in each trimester) were examined for blood lipid and glucose parameters at approximately 10 years of age. The authors found an association between second and third trimester phthalate levels and lower glucose and higher triglyceride concentrations in boys, respectively [69]. These results suggest a gender-specific relationship with phthalate exposure that could be related to metabolic impairment. In an attempt to discover the connection between DEHP substitutes and insulin resistance, one spot urine sample was collected from 356 fasting adolescents (12–19 years old). In addition to finding a correlation with DEHP as expected, insulin resistance was also related to DINP concentrations [70]. On the other hand, no connection between urinary phthalates and insulin resistance was found in a population of 107 Danish children (mean age of 12 years) [71]. Nevertheless, a different study has shown that age and gender may play a role in the correlation between phthalate exposure and insulin resistance. In a young Taiwanese population, from adolescents to young adults, a link between elevated urinary levels of MEHP and incidence of insulin resistance was shown to occur in young adults (20–30 years old), but not in adolescents (12–19 years old). Moreover, in the same age range, MEHP was also related to decreased testosterone levels in males, suggesting that testosterone levels are inversely related to insulin resistance [72].

Analyzing a broader age range (12–79 years old), participants were asked to provide a one-time mid-stream urine sample for phthalate measurement, and one blood sample for insulin and glucose parameters. Associations were found between MBzP, MiBP, MCPP, MEHP, MEHHP, and ∑DEHP with HbA1c levels, and between DEHP metabolites with higher amounts of insulin, insulin resistance and fasting glucose, reduced glucose control, and β-cell function, suggesting an involvement of phthalates in pre-diabetes [73]. Considering the straight connection between diabetes mellitus and obesity, Dirinck et al. analyzed the correlation between urinary phthalate concentrations from a 24 h urine sample and glucose metabolism in an obese/overweight population (123 adults, aged between 18 and 84 years). There was an association between phthalate metabolites and several metabolic biomarkers related to insulin; specifically, there was a positive association with resistance and impaired β-cell function, and a negative one with insulin sensitivity, even after correction for BMI. However, opposite to the study conducted by Dales et al., there was no association with HbA1c levels. The results from this study suggest phthalates as being higher risk factors for diabetes than obesity [74]. There was also a relationship between increased urinary levels of phthalate metabolites and the incidence of T2DM, when examining a much larger population sample (*n* = 3781), and, despite being separated by gender, no association was found between male and female results [75]. On the other hand, in a Chinese case-control study, differences among gender, age, and BMI were found. A total of 500 participants with and without T2DM provided one spot urine sample, and T2DM participants had higher and more significant levels of MEHHP, MEOHP, MEHP, MCPP, MiBP, MMP, and ∑DEHP and decreased levels of MECPP and MCMHP. When stratified, the associations between phthalate metabolites and T2DM, HbA1c levels, and fasting glucose were more prominent for participants younger than 55 years old, with BMI inferior to 25 Kg/m^2^, and males older than 55 years old, respectively [76]. Similarly, in a population sample of 2330 participants from Shanghai (mean age of 53 years), Dong and colleagues also found a significant association between urinary phthalate levels and T2DM in men only, specifically, MEOHP, MEHHP, and MECPP [77]. Using men only, a case-control study of 100 diabetic and 50 non-diabetic participants found higher concentrations of MEP, MEOHP, and MBP in the cases of T2DM, with MEP and MBP being related to HOMA-IR and C-peptide, which are linked to insulin resistance [78]. In accordance with these results, an Australian cohort of 1504 men (39–84 years old) also found an association between phthalate exposure and T2DM [79]. These previous studies show the importance and the need for a sex-specific assessment across all ages, considering that phthalates are known to interact with androgen and estrogen receptors.

Nevertheless, despite gender-related differences, some older epidemiological studies have also shown a relationship between exposure to phthalates and T2DM in women. Upon investigating different populations, increased urinary levels of MBP, MiBP, MBzP, MCPP, ∑DEHP, and ∑DBP were found to be related to T2DM [80,81,82]. In a different perspective, 618 women provided urine samples in the second and third trimesters of pregnancy, which were compared with metabolic parameters measured in blood several years later. There was a positive association between urinary phthalate (mainly MECPTP and DBP) levels and insulin resistance, considering the high amounts of plasma glucose, insulin, HOMA-IR, and HbA1c% [38].

In an attempt to understand the role of metabolism in the development of T2DM due to phthalate exposure, a case-control study of 60 diabetic and 60 non-diabetic participants was performed by Duan et al.. From the fasting blood samples collected, metabolites and metabolic pathways were investigated between cases and controls and compared with urinary phthalate concentrations. Overall, there was an association between phthalate metabolites and galactose, amino acid, and pyrimidine metabolism in T2DM subjects [83].

Considering the already demonstrated effects of phthalates on T2DM, a different epidemiologic study aimed to understand whether diuretic compounds had the ability to increase the urinary excretion of phthalate metabolites. In a randomized clinical trial of 30 diabetic and hypertensive patients, half were treated with a SGLT2 inhibitor and the other half with a thiazide for 4 weeks; the results showed a higher urinary excretion of DEHP metabolites after both treatments, thus reducing the time of exposure to phthalates. Therefore, it concluded that the use of these two classes of drugs can reduce the toxicity caused by these contaminants [84].

Overall, as is summarized in Table 2, the majority of the studies presented show an association between the exposure to phthalates, mostly DEHP and its metabolites, and the occurrence of T2DM, from children to elderly people, but there are still some inconsistencies in the results. Considering the epidemiological nature of the studies, such variances are expected, particularly due to genetic variability among the population samples, and, although the associated mechanisms are already beginning to be unraveled, more studies are needed to understand how phthalates affect the development of T2DM. For this, new experimental studies should be performed, and, considering the ubiquitousness of phthalates, extreme care must be taken during laboratory handling, particularly with regard to the material used (preferably glass or phthalate-free plastics), and blanks must be performed in order to eliminate/avoid the background exposure.

#### 4.3.2. Experimental Studies

Despite the scarcity of experimental studies regarding the effect of prenatal exposure on the development of GDM, as previously mentioned, there is more information on the metabolic effects that this type of exposure has on offspring. Three different studies related gestational DEHP exposure to glucose parameters in adults of the F1 generation. To achieve the goals, female Wistar rats were exposed to different concentrations of DEHP (1, 10 and 100 mg/Kg/day) from gestational day (GD) 9 to GD 21 [85] and to postnatal day (PND) 21 [86,87]. In the first study, Rajesh et al. found that DEHP induced changes in the expression of genes related to insulin gene transcription and a glucose sensing mechanism, culminating in β-cell dysfunction [85]. The other two investigations also evaluated the lactation period and analyzed only the effects observed in adult male offspring. The results showed that DEHP exposure led to impaired regulation of the GLUT2 gene and insulin signal transduction, leading to decreased glucose tolerance, insulin resistance, and hyperglycemia [86,87]. All the events reported from these three studies may lead to T2DM in offspring.

In a different perspective, male Balb/c mice were exposed to three different doses of DBP for 7 weeks, in which the highest DBP dose led to decreased insulin secretion and glucose intolerance. Moreover, when using STZ and a high-fat diet to induce T2DM, the exposure to DBP worsened the affected parameters and induced insulin resistance and T2DM-related organ lesions. The T2DM mouse model also presented a decreased PI3K/AKT signaling pathway and increased pancreatic GLUT2, which may be implicated in the DBP mechanism [88].

Two studies from the same research group evaluated the effects of DEHP in adolescent (3-week-old) female [89] and male [90] ICR mice with and without T2DM. Upon the administration of four different concentrations of DEHP for 3 weeks, glucose and lipid parameters, as well as cardiovascular risk were analyzed in the different study groups. The results showed that both T2DM male and female mice were more susceptible to DEHP exposure than normal mice; however, T2DM female mice proved to be more sensitive than their male counterparts, with an increased risk of suffering from T2DM, metabolic and cardiovascular disorders, and hepatotoxicity. Moreover, it was also suggested that DEHP activates Jun-N-terminal kinase (JNK), promoting the apoptosis of hepatic cells and the inhibition of insulin sensitivity, which may lead to metabolic disorders [89,90]. The results of these studies also allowed the authors to assume the gender differences caused by the exposure to DEHP with the incidence in female mice, which are in accordance with other reports; however, the epidemiological studies relating sex-specific differences tend to show a higher incidence in men [69,72,76,77].

Upon the exposure of the pancreatic β-cell line (INS-1) to a range of concentrations (0.001–10 µM) of MEHP and MBP for 24, 48, and 72 h, there was cell viability decrease and oxidative stress increase with mRNA expression changes for genes related to pancreatic β-cell function and apoptosis. These results imply that MEHP and MBP might affect β-cell function, which may lead to insulin resistance and consequent T2DM [91].

As was previously stated, the study conducted by Weldingh and co-workers reported an inferior potency of MEHP, MBP, and MiBP compared to BPA in affecting insulin secretion in INS-1E cells. However, it is important to note that phthalates in the serum are found in much higher concentrations than BPA, and so a new approach closer to real human exposure is needed [56]. In a different study using human pancreatic β-cells (1.1B4), a 24 h exposure to low concentrations of MEP (1–1000 nM) led to increased insulin secretion, possibly involving ERα, PPARγ, and PDX-1 (pancreatic and duodenal homeobox 1), which are related to β-cell function and survival [92]. Al-Abdulla and colleagues also demonstrated that exposure to DEHP led to impaired insulin secretion in both human and murine pancreatic β-cells [93].

Several authors have been investigating the role of oxidative stress in phthalate-induced T2DM. In an in vivo study, male Swiss albino mice (8-week-old) were treated with DEP for 3 months, after which serum, liver, and epididymal adipose tissue were removed for further analysis. Besides concluding that this chronic low-level exposure to DEP induced impaired insulin signaling in both hepatocytes and adipocytes, the authors also discovered a great increase in NOX2 (NADPH oxidase 2), which is involved in the generation of ROS [94]. Differentiated human preadipocytes were used by Schaffert et al. to analyze the effects of 20 plasticizers in PPARγ. In preadipocytes, DINP and DPHP (DEHP substitutes) metabolites activated PPARγ, inducing lipid accumulation and adipogenesis, while in mature adipocytes these compounds promoted lipid storage, oxidative stress, and impaired adipokine release related to insulin resistance [95]. Two in vitro studies on the same cell line (INS-1) found that both DEHP and DBP exert their adverse effects through oxidative stress. Specifically, DEHP acts in the lysosome–mitochondrial axis pathway, increasing ROS production and leading to DNA damage and p53 and ATM activation [96]. Additionally, on the other hand, DBP altered PDX-1 and GLUT2 levels, leading to reduced insulin synthesis and secretion through the mitochondrial apoptotic pathway and oxidative stress [97].

Viswanathan and collaborators analyzed how DEHP and MEHP affected GLUT4 in a cell model of the skeletal muscle (L6 myotubes). After the incubation of the cells with 50 and 100 µM DEHP and MEHP (24 h), the authors observed changes in GLUT4 levels and translocation, as well as in insulin signaling molecules [98]. Similarly, GLUT4 was also shown to be affected by DEHP, either in in vivo or in vitro experiments. Moreover, Sprague Dawley rats exposed to DEHP exhibited liver damage, glucose, and insulin tolerance, while in a human hepatocyte cell line (L02), DEHP interacted with PPARγ, increasing ROS levels [99]. These studies emphasize the role of PPARγ and oxidative stress in the development of T2DM induced by phthalates.

Some investigations have also demonstrated a protective or reversible role of certain molecules or compounds towards damaging phthalate effects. In the study of Deng and colleagues, when a selective insulin receptor activator, demethylasterriquinone B1 (DMAQ-B1), was administered to mice, there was a decrease in the adverse effects of DBP regarding insulin deficiency and resistance [88]. Additionally, according to She et al., pyrroloquinoline quinone (PQQ)—a compound with anti-inflammatory, anti-oxidative, hepato-, and cardioprotective properties—has the capacity to protect INS-1 cells from the adverse effects promoted by DEHP [96]. In a study that combined computational analysis with in vivo experiments, after finding that the conjoint action of DEHP, DBP, and BPA led to T2DM in rats through oxidative stress and apoptosis, a protective role of a mixture of probiotics, regarding redox properties in the pancreas, was also observed [100]. All these experimental studies are summarized in Table 3.

#### 4.3.3. Possible Mechanisms

As previously stated, some hypotheses have emerged for the phthalates’ mechanism of action, both in epidemiological and experimental studies. So far, oxidative stress has been the most studied and with more positive evidence, but inflammatory markers, impaired adiponectin, and β-cell dysfunction have also been gaining attention.

Pancreatic β-cell dysfunction is one of the main causes for T2DM development, and some studies have shown that phthalates affect these cells through different pathways [85,91,92]. Maternal exposure to DEHP was shown to promote disrupt β-cell function in the rat offspring by affecting the glucose sensing mechanism and insulin gene transcription [85]. On the other hand, through the activation of ERα, PPARγ, and PDX-1, MEP increases insulin secretion, which, as previously mentioned, with time will progress to the failure and loss of the pancreatic β-cells [92]. This is in accordance with previous mentioned studies, since estrogens are related to insulin resistance, and thus phthalates can affect insulin signaling through Erα-mediated pathways [30]. In addition, MEHP and MBP were shown to affect the expression of β-cell-related genes and promote oxidative stress [91]. In fact, oxidative stress has been suggested as one of the possible mechanisms for the development of T2DM by exposure to phthalates, either in experimental or epidemiological studies [94,96,97]. DEP and DEHP are involved in the generation of ROS by increasing NOX2 [94] and MDA levels [96]. In the mechanism proposed by She et al., DEHP promoted lysosomal disruption in INS-1 cells, decreasing mitochondrial membrane potential, and thus increasing ROS production and p53 and ATM activation, which are related to DNA damage [96].

Other molecular pathways have been implicated and suggested as mechanisms for insulin resistance and T2DM. DEHP was shown to activate JNK, affecting Bcl-2 and Bax, leading to apoptosis and the inhibition of the insulin sensitivity of mice hepatic cells [89,90]. Moreover, both DEHP and DBP inhibited the PI3K/AKT signaling pathway and led to impaired glucose transporters (GLUT2 and GLUT4), resulting in decreased glucose tolerance, insulin resistance, and hyperglycemia [86,87,88,89,97,98]. Moreover, there seems to be a sex-specific effect of DEHP, since higher risks for T2DM were demonstrated in female mice [89].

Phthalates are considered as peroxisome proliferator activators, and many of their adverse effects may occur through the PPARs [101]. In human experimental studies, the mechanism for phthalate-induced insulin resistance seems to involve the activation of PPARγ and oxidative stress. DEHP and its substitutes, DINP and DPHP, promoted the activation of PPARγ in human preadipocytes [95], while in hepatocytes only DEHP activated PPAR [99]. Moreover, in both hepatocytes and adipocytes, the compounds induced oxidative stress, thus disturbing lipid and glucose metabolism leading to insulin resistance [95,99].

It is known that oxidative stress, adiponectin, and inflammatory cytokines play an important role in the pathophysiology of T2DM, and experimental studies have indicated a connection between the exposure to phthalates and these parameters [95,96,97]. Thus, an epidemiological study aimed to analyze phthalate concentrations in the urine of diabetic subjects and biomarkers for oxidative stress (MDA), adiponectin, and inflammation (TNF-α). All phthalates measured were correlated with MDA, and MMP was positively and negatively correlated with TNF-α and adiponectin, respectively. Moreover, participants with a higher BMI presented an inverse association between ∑DEHP and adiponectin, and a proportional relationship regarding ∑DEHP and MEHP, and TNF-α [102]. A link between increased phthalate exposure, oxidative stress parameters (8-OHdG, 8-PGF2α,15-PGF2α, MDA), and a higher T2DM incidence was also found in two different populations [103,104], through a mechanism that may be mediated by γ-glutamiltransferase [104]. With the results of these studies, we can state that insulin resistance due to phthalate exposure might involve oxidative stress, adiponectin, and inflammatory factors, thus being implicated in the development of T2DM.

## 5. Conclusions

The prevalence of diabetes mellitus is quite high all over the world and is set to increase further in the coming years [8]. Along with this, phthalate contamination has been continuously increased due to their ubiquitousness and widespread use [105]. Thus, with this review, we aimed to explore the potential relationship between phthalate exposure and diabetes, reporting on the most recent evidence.

GDM and T2DM have been extensively analyzed regarding phthalate exposure. As pregnant women are considered a more susceptible population, several studies, mainly epidemiological ones, have been evaluating the effect of phthalates on GDM, looking at both its severity during pregnancy and the adverse effects that it may have in the future on the woman and her offspring. Some of the mentioned studies present contradictory results, with negative, weak, and positive associations between the metabolites of several phthalates and diabetes. Many consider the first trimester as the most sensitive period for environmental contamination, especially for the fetus, but, even so, it is necessary to understand the trimester-specific differences and evaluate the possible effects in each of the stages, both for the mother and for the children. Regarding the effects on T2DM, several studies, from children to elderly, have been conducted. In general, they report a possible correlation with phthalate exposure, with these contaminants affecting several glucose metabolism parameters (glucose and insulin levels, insulin tolerance, HOMA-IR, HbA1c, β-cell function) that will lead to insulin resistance.. Some findings also suggested gender-specific differences, in which epidemiological studies seem to show a greater association with males, while animal studies report a greater incidence in females. These outcomes make it difficult to extrapolate the results from the animals to human beings, and indicate the need to carry out more studies in order to understand the connection with the gender. However, considering that phthalates are endocrine disruptors with the ability to act on hormones and their receptors, and that hormonal levels change across age groups, it makes perfect sense that these differences exist. Despite the great scarcity of studies regarding T1DM, the epidemiological and experimental ones performed so far have also suggested a possible association with phthalates.

It is important to emphasize that several possible mechanisms have been suggested, and, despite having different etiologies, most of these suggestions are transversal to all types of diabetes mellitus addressed in this article. So, from the information retrieved, phthalates may lead to insulin resistance and consequent diabetes mellitus through oxidative stress, the activation of different hormone receptors (PPAR and ER), and impaired inflammatory factors. Nevertheless, the need to carry out further studies in order to unravel the mechanisms involved still remains.

Although most studies point to a possible link between the exposure to phthalates and the development of DM, some of them showed no interaction. These differences might have several explanations: (1) the variation in population samples, either regarding the number of participants or different racial/ethnicity characteristics; (2) the biological samples collected, considering that blood samples present more disadvantages than urine, mainly regarding the possible contamination, limited volume, and invasiveness, and also the quantity of samples collected throughout pregnancy, since multiple samples would be more accurate for evaluation; (3) potential confounding results regarding EDC exposure, since people are not only exposed to phthalates but also to mixtures of phthalates and/or mixtures of several EDCs; and (4) the different criteria for diabetes diagnosis throughout all the studies, from GDM to T2DM.

Regarding the epidemiological studies, attention must be made to the study design, with all of them being subject to limitations and advantages. Cohort studies, either prospective or retrospective, have the advantage of assessing causality and multiple outcomes, but require a large number of participants and longer follow-ups, in addition to being expensive studies to conduct. Some disadvantages of case-control studies are the susceptibility to recall information bias and the difficulty to validate information, but, on the other hand, they represent great strategies for investigating rare outcomes, have short follow-up times, and are relatively inexpensive. Lastly, cross-sectional surveys are subject to the time of data collection, and causality cannot be inferred [106]. Many experimental studies have been performed in this manner and virtually all of them suggest a link between phthalates and DM. However, most of them were performed in animals (mice or rat) and so the results cannot always be extrapolated to humans, and in vitro studies fail to replicate the whole organism. Nevertheless, experimental studies represent one of the most valuable tools in research. Thus, they are considered as the best strategy to obtain more reliable results, and the best way to understand how environmental contaminants affect human health is to combine prospective and experimental studies [107].

Overall, some studies suggest that exposure to phthalates may influence glucose- and insulin-related parameters, culminating in the development of diabetes mellitus, from pregnant women to elderly people. So, in addition to the need for further studies, specifically a combination of prospective and experimental, as mentioned, it is important that the population takes preventive measures regarding their daily exposure to EDCs in order to improve their health and quality of life [108].

## Figures and Tables

**Table 1:** Summary of the epidemiologic and experimental studies regarding phthalate outcomes in gestational diabetes mellitus.

Study Type	Phthalate	Biological Sample		Population			Findings	Ref
		Matrix	Quantity	Country	Size	Age		
Cohort	MEP, MBP, MCPP, ∑DEHP	Urine Blood	4 Gestational weeks 10, 18, 26, 35	USA	350	31.9 (mean)	- Positive association between MEP and GWG. - Negative association between the following: MBP and excessive GWG; MCPP and blood glucose; ∑DEHP and IGT.	[27]
Cohort	MiBP, MHBP MBP, MCNP, MCPP	Urine	4 Gestational weeks 10, 18, 26, 35	USA	606	33.5 (mean)	- Positive association between MiBP, MHBP, and IGT. - Negative association between MBP, MCNP, MCPP and GDM, IGT.	[28]
Cohort	-	Urine	4 Gestational weeks 10, 18, 26, 35	USA	350	31.9 (mean)	No association	[29]
Cohort	MEP, MBP, MCOP, MCPP	Urine	2 1st and 3rd trimesters	USA	705	31 (mean)	- Positive association between the following: MEP and GDM; MBP, MCOP and IGT. - Negative association between MCPP and GDM.	[30]
Cohort	MEP, MiBP	Urine	3 Each trimester	USA	245	35.3 (mean)	- Positive association between MEP and glucose levels. - Negative association between MiBP and glucose levels.	[31]
Cohort	Phthalates	Blood	1 Late 2nd trimester	USA	233	35.4 (mean)	- Indirect association between MEP and glucose levels.	[19]
Cohort	MBP, MMP, MEOHP, MEHHP	Urine Serum	3 Each trimester	China	3269	24–35	- Positive association with increased blood glucose in the 1st trimester.	[32]
Case-control	MEHP, MMP, MEP, MiBP, MECPP, MEOHP	Urine Blood	1 Early 3rd trimester	China	200	32 (mean)	- Higher MEHP levels in GDM cases. - Positive association with fasting blood glucose and insulin, and insulin resistance index.	[33]
Case-control	MnOP, MBzP, MEOHP, MECPP	Urine	1 1st trimester	China	676	20–35	- Positive association with GDM.	[34]
Case-control	MBP, MiBP	Serum	1 Childbirth	China	201	22–43	- Positive association with 2 h glucose levels.	[35]
Case-control	MiBP, MEHP, MCOP	Serum	1 Gestational weeks 10–17	UK	232	≈33 (mean)	- Positive association between MiBP and GDM. - Positive association between MEHP, MCOP and glucose levels in women w/o GDM.	[36]
Case-control	MBzP, MBP, MEHP, MiBP	Urine Blood	1 2nd trimester	Mexico	40	24–45	- Positive association with miRNA in GDM.	[37]
Cohort	MECPTP, ∑DBP	Urine Blood	2	Mexico	618	27.3 (mean)	- Positive association with glucose and insulin levels, insulin resistance, and HbA1c.	[38]
			- 2nd and 3rd trimesters - After 4–8 years after					
Cross-sectional	MBP, MiBP, MEHP	Meconium	1 Childbirth	China	251	29 (mean)	- Positive association with GDM for mothers of male fetuses.	[39]
Cohort	Phthalate mixtures	Urine Blood	2 Late 2nd and 3rd trimesters	USA	1018	26.4 (mean)	- Negative association with pCRH levels in women with GDM.	[40]
Cohort	-	Urine	1 1st trimester	Canada	1274	≥18	No association	[41]
Cohort	-	Urine	1 1st or 2nd trimesters	USA	72	22 (mean)	No association	[42]
Cohort	-	Urine	2 Late 1st and 2nd trimesters	USA	415	18–45	No association	[43]

GWG—gestational weight gain; IGT—impaired glucose tolerance; HbA1c—glycosylated hemoglobin; pCRH—placental corticotropin-releasing hormone.

**Table 2:** Summary of the epidemiologic studies regarding phthalate outcomes in type 2 diabetes mellitus.

Study Type	Phthalate	Biological Sample	Population				Findings	Ref
			Country	Size	Age	Gender		
Cohort	HMWP LMWP	- Urine: 3 (each trimester of pregnancy) - Blood: 1 (child)	Netherlands	757	Mother: 31 Child: 9.7 (mean)	M/F	Sex specific effects for boys:	[69]
							- Negative association glucose levels in the 2nd trimester; - Positive association with triglyceride levels in the 3rd trimester.	
Cross-sectional	DEHP, DINP	- Urine: 1 spot sample	USA	356	12–19	M/F	- Positive association with insulin resistance.	[70]
Cross-sectional	-	- Urine: 2 first morning and 1 24 h samples - Blood: 1	Denmark	107	12 (mean)	M/F	No association	[71]
Cross-sectional	MEHP	- Urine: 1 first morning - Blood: 1	Taiwan	786	12–30	M/F	In young adults (20–30 years old) group:	[72]
							- Positive association with insulin resistance; - Negative association with testosterone levels in males.	
Cross-sectional	MBzP, MiBP, MCPP, MEHP, MEHHP, ∑DEHP	- Urine: 1 mid-stream - Blood: 1	Canada	2119	12–79	M/F	- Positive association between the following: MBzP, MiBP, MCPP, MEHP, MEHHP, ∑DEHP and HbA1c, DEHP and insulin, insulin resistance, fasting glucose. - Negative association between DEHP and glucose control, β-cell function.	[73]
Cross-sectional	Phthalate metabolites	- Urine: 1 24 h sample - Blood: 1	Belgium	123	18–84	M/F	- Positive association with insulin resistance and impaired β-cell function. - Negative association with insulin sensitivity.	[74]
Cross-sectional	MBzP, MBP, MCPP, DEHP, MEOHP, MEHHP	- Urine: 1 spot sample	South Korea	3781	19- ≥ 70	M/F	- Positive association with T2DM incidence.	[75]
Case-control	MEHHP, MEOHP, MEHP, MCPP, MiBP, MMP, ∑DEHP, MECPP, MCMHP	- Urine: 1 spot sample	China	500	Case: 58 Control: 51 (mean)	M/F	- Positive association with T2DM incidence, except MECPP, MCMHP, with lower levels. Association between phthalate metabolites and T2DM for younger than 55 years old, HbA1c for BMI inferior to 25 Kg/m^2^, Fasting glucose for males older than 55 years old.	[76]
Cross-sectional	MEOHP, MEHHP, MECPP	- Urine: 1 spot sample	China	2330	53 (mean)	M/F	Sex specific effects for men:	[77]
							- Positive association with T2DM incidence.	
Case-control	MEP, MEOHP, MBP	- Urine: 1 - Blood: 1	Saudi Arabia	150	45 (mean)	M	- Positive association with T2DM incidence, HOMA-IR, C-peptide.	[78]
Cross-sectional	Total phthalates	- Urine: 1 first morning sample - Blood: 1	Australia	1504	39–84	M	- Positive association with T2DM incidence.	[79]
Case-control	MBzP, MEOHP, MEHHP, MECPP	- Urine: 1 first morning sample	Mexico	221	Case: 60.5 Control: 52.4 (mean)	F	- Positive association between DEHP metabolites and T2DM. - Negative association between MBzP and T2DM.	[80]
Cross-sectional	MBP, MBzP, MiBP, MCPP, ∑DEHP	- Urine: 1 spot sample	USA	2350	20–79	F	- Positive association with T2DM incidence.	[81]
Case-control	∑DEHP, ∑DBP	- Urine: 1 spot sample	USA	1941	Premenopausal: 45.6 Postmenopausal: 65.6 (mean)	F	- Positive association with T2DM in the premenopausal group.	[82]
Cohort	MECPTP, DBP	- Urine: 2 (2nd and 3rd trimesters of pregnancy) - Blood: 2 (4–5 and 6–8 years post-delivery)	Mexico	618	27.7 (mean)	F	- Positive association with insulin resistance.	[38]
Case-control	∑DEHP, MCPP, MiBP, MMP	- Urine: 1 - Blood: 1	China	120	56 (mean)	M/F	- Positive association with galactose, amino acid, pyrimidine metabolism in T2DM subjects.	[83]
Clinical trial	DEHP metabolites	- Urine: 2 24 h samples (before and after treatment)	Italy	30	60 (mean)	M/F	- Increased urinary excretion of DEHP metabolites after treatment with a SGLT2 inhibitor or a thiazide.	[84]

M—male; F—female.

**Table 3:** Summary of the experimental studies regarding phthalate outcomes in type 2 diabetes mellitus.

Study Type	Animal/Cell Type	Phthalate	Treatment		Findings	Ref
			Dose/Concentration	Duration		
In Vivo	Pregnant Wistar rats	DEHP	1, 10, 100 mg/Kg/day	GD 9 to GD 21	Changes in expression of insulin gene transcription and glucose sensing mechanism-related genes leading to β-cell dysfunction in F1 offspring.	[85]
In Vivo	Pregnant Wistar rats	DEHP	10, 100 mg/Kg/day	GD 9 to PND 21	Impaired insulin signal transduction and glucoregulatory events in F1 male offspring leading to decreased glucose tolerance, IR, and hyperglycemia.	[86]
In Vivo	Pregnant Wistar rats	DEHP	10, 100 mg/Kg/day	GD 9 to PND 21	Impaired regulation of GLUT2 gene and epigenetic changes in IR and GLUT2 gene promoters.	[87]
In Vivo	Male Balb/c mice (5–6 weeks old)	DBP	0.5, 5, 50 mg/Kg/day	7 weeks	Highest DBP dose decreased insulin secretion and glucose intolerance. T2DM mouse model: IR, organ lesions, decreased PI3K/AKT signaling pathway, increased pancreatic GLUT2. Administration of selective insulin receptor activator (DMAQ-B1) decreased the adverse effects on insulin deficiency and resistance.	[88]
In Vivo	Female ICR mice with and w/o T2DM (3 weeks old)	DEHP	0.18, 1.8, 18, 180 mg/Kg/day	3 weeks	Female T2DM mice more susceptible to DEHP than male and normal mice. Activation of JNK and impaired insulin sensitivity in the liver.	[89]
In Vivo	Male ICR mice with and w/o T2DM (3 weeks old)	DEHP	0.18, 1.8, 18, 180 mg/Kg/day	3 weeks	Impaired endocrine and metabolic functions. Increased IR. T2DM mice more susceptible to DEHP than normal mice.	[90]
In Vitro	Rat pancreatic β-cell line (INS-1)	MEHP, MBP	0.001–10 µM	24, 48, 72 h	Decreased cell viability. Increased oxidative stress. Gene expression changes related to pancreatic β-cell function and apoptosis.	[91]
In Vitro	Rat pancreatic β-cell line (INS-1E)	MEHP, MBP, MiBP	5, 50, 500 µM	2, 24, 48, 72 h	Decreased potency of phthalates, compared to BPA, affected insulin secretion.	[56]
In Vitro	Human pancreatic β-cells (1.1B4)	MEP	1–1000 nM	24 h	Increased insulin secretion, possibly involving ERα, PPARγ, and PDX-1.	[92]
In Vitro	Murine pancreatic β-cell line (MIN6) Human pancreatic β-cell line (EndoC-βH1)	DEHP	100 pM-10 µM	24, 48, 72 h, or 7 days	Impaired insulin secretion in both cell lines.	[93]
In Vivo	Male Swiss albino mice (8-week-old)	DEP	1, 10 mg/Kg.bw/day	3 months	Chronic exposure leading to impaired insulin signaling in hepatocytes and adipocytes. Increased NOX2 levels involved in the generation of ROS.	[94]
In Vitro	Differentiated human preadipocytes of the Simpson-Golabi-Behmel syndrome (SGBS) cell line	DINP, DPHP	0.01–100 µM	Preadipocytes: 16 days Mature adipocytes: 8 days	Activation of PPARγ in preadipocytes leading to lipid accumulation and adipogenesis. Lipid storage, oxidative stress, and impaired adipokine release in mature adipocytes.	[95]
In Vitro	Rat pancreatic β-cell line (INS-1)	DEHP	MTT: 0–1600 µM Other experiments: 0–400 µM	1 h or 24 h	Involvement of the lysosome–mitochondrial axis pathway through oxidative stress and p53 and ATM activation. Protective effect of PQQ.	[96]
In Vitro	Rat pancreatic β-cell line (INS-1)	DBP	MTT: 15, 30, 60, 120 µM Other experiments: 15, 30, 60 µM	24 h	Altered PDX-1 and GLUT-2 levels, which reduced insulin synthesis and secretion through mitochondrial apoptotic pathway and oxidative stress.	[97]
In Vitro	Rat skeletal muscle model (L6 myoblast cells)	DEHP, MEHP	50, 100 µM	24 h	Changes in GLUT4 levels and translocation. Changes in insulin signaling molecules.	[98]
In Vivo In Vitro	Male Sprague Dawley rats Human hepatocyte cell line (L02)	DEHP	In Vivo: 0.05, 5, 500 mg/Kg.bw In Vitro: 5, 10, 25, 50, 100 µM	In Vivo: 15 weeks In Vitro: 24, 48 h	In Vivo: liver damage, glucose and insulin tolerance, reduced insulin receptor and GLUT4 protein expression. In Vitro: interaction with PPARγ, increased ROS levels, reduced insulin receptor and GLUT4 protein expression.	[99]
In Silico In Vivo	Male albino rats	DEHP, DBP, (BPA)	50 mg/Kg.bw/day (25 mg/Kg.bw/day)	28 days	Joint action of DEHP, DBP, and BPA led to T2DM through oxidative stress and apoptosis. Protective role of probiotic mixture regarding redox properties in the pancreas.	[100]

GD—gestational day; PND—postnatal day; IR—insulin resistance; DMAQ-B1—demethylasterriquinone B1; JNK—Jun-N-terminal kinase; ERα—estrogen receptor alpha; PPARγ—peroxisome proliferator-activated receptor gamma; PDX-1—pancreatic and duodenal homeobox 1; NOX2—NADPH oxidase 2; ROS—reactive oxygen species; PQQ—pyrroloquinoline quinone.

## Abbreviations

∑DBP	Sum of DBP metabolites
∑DEHP	Sum of DEHP metabolites
1.1B4	Human pancreatic β-cells
8-OHdG	8-hydroxy-2′-deoxyguanosine
8-PGF2α	8-iso-prostaglandin F2α
ATM	Ataxia-telangiectasia mutated
Bax	Bcl-2-associated X protein
BBzP	Butylbenzyl phthalate
Bcl-2	B-cell lymphoma 2 anti-apoptotic protein
BMI	Body mass index
BPA	Bisphenol-A
DBP	Di-butyl phthalate
DEHP	Di-(2-ethylhexyl) phthalate
DEP	Diethyl phthalate
DiBP	Di-isobutyl phthalate
DiDP	Diisodecyl phthalate
DiNP	Diisononyl phthalate
DMP	Dimethyl phthalate
DMAQ-B1	Demethylasterriquinone B1
DnOP	Di-n-octyl phthalate
DPHP	Di(2-propylheptyl) phthalate
EDC	Endocrine disruptor compound
ER	Estrogen receptors
FoxM1	Forkhead box protein M1
GD	Gestational day
GDM	Gestational diabetes mellitus
GLUT	Glucose transporter protein
GWG	Gestational weight gain
HbA1c	Glycosylated hemoglobin
HMW	High molecular weight phthalates
HOMA-IR	Homeostasis model assessment-estimated insulin resistance
IDF	International diabetes federation
INS-1E	Rat pancreatic β-cell line
JNK	Jun-N-terminal kinase
LMW	Low molecular weight phthalates
MBP	Mono-n-butyl phthalate
MBzP	Mono-benzyl phthalate
MCMHP	Mono-(2-carboxymethyl-hexyl) phthalate
MCNP	Mono-(carboxy-isononyl) phthalate
MCOP	Mono-(carboxy-isooctyl) phthalate
MCPP	Mono-(3-carboxypropyl) phthalate
MDA	Malondialdehyde
MECPP	Mono-(2-ethyl-5-carboxypentyl) phthalate
MECPTP	Mono-2-ethyl-5-carboxypentyl terephthalate
MEHHP	Mono-(2-ethyl-5-hydroxyhexyl) phthalate
MEHP	Mono-(2-ethylhexyl) phthalate
MEOHP	Mono-(2-ethyl-5-oxohexyl) phthalate
MEP	Mono-ethyl phthalate
MiBP	Mono-isobutyl phthalate
MHBP	Mono-(3-hydroxybutyl) phthalate
MMP	Mono-methyl phthalate
MnOP	Mono-n-octyl phthalate
NOD	Non-obese diabetic
NOX2	NADPH oxidase 2
p53	Tumor protein P53
pCRH	Placental corticotropin-releasing hormone
PDX-1	Pancreatic and duodenal homeobox 1
PI3K/AKT signaling pathway	Phosphoinositide 3-kinase/Akt
PND	Postnatal day
PPAR	Peroxisome proliferator-activated receptors
PQQ	Pyrroloquinoline quinone
pSTAT1	Phosphorylated signal transducer and activator of transcription 1
ROS	Reactive oxygen species
SGLT2	Sodium-glucose transport protein 2
STZ	Streptozotocin
T1DM	Type 1 diabetes mellitus
T2DM	Type 2 diabetes mellitus
TNF-α	Tumor necrosis factor
